# Effect of partial replacement of potassium sources combined with cobalt on wheat (*Triticum aestivum L*.) growth and yield parameters

**DOI:** 10.1038/s41598-026-63334-0

**Published:** 2026-08-18

**Authors:** Nadia Gad, M. E. Fekry Ali, Hanan G. Ismail, A. A. Hafez, Ahmed Fathy Yousef, Eman Ali Abd Elrahman

**Affiliations:** 1 Plant Nutrition Department, National Research Centre, Cairo, Egypt; 2 Department of Soil and Agricultural Chemistry, Faculty of Agriculture (Saba Basha), Alexandria University, Alexandria, 21531 Egypt; 3 Department of Agronomy, Faculty of Agriculture, Al-Azhar University (Assiut branch), Assiut, 71524 Egypt; 4 Horticulture Department, Agriculture College, Al-Azhar University (Assiut Branch), Assiut, 71524 Egypt

**Keywords:** Wheat (*Triticum aestivum*), Potassium sulfate, Potassium chloride, Cobalt supplementation, Grain quality, Nutrient uptake, Yield performance, Plant sciences, Environmental sciences

## Abstract

This study evaluated the interactive effects of potassium (K) fertilizer sources and cobalt (Co) supplementation on the growth, yield, and grain quality of wheat (*Triticum aestivum* cv. Giza-95) under arid soil conditions in El-Nubaryia, Egypt. A split-split-plot field experiment was conducted over two consecutive seasons (2021/22 and 2022/23), testing five K-fertilization regimes (100% K_2_SO_4_, 75% K_2_SO_4_ + 25% KCl, 50% K_2_SO_4_ + 50% KCl, 25% K_2_SO_4_ + 75% KCl, and 100% KCl) with or without 10 mg L^−1^ cobalt. Results showed that both K source and Co significantly affected plant growth, yield traits, nutrient uptake, and biochemical constituents of wheat grains. Cobalt consistently increased plant height, tillering, spike number, spike length, and dry matter accumulation, while also enhancing grain filling, straw yield, and grain yield (up to 9.35 t ha^−1^ with 100% K_2_SO_4_ + Co). Grain nutritional quality improved markedly, with cobalt raising protein (by 3.5%), carbohydrates, starch, and soluble sugars (by up to 18.3%), while also increasing Zn, Mn, Cu, and Co concentrations in grains. All mixing ratios of potassium sulfate and potassium chloride produced better results than potassium chloride alone. Conversely, increasing KCl proportion reduced vegetative growth, yield, and nutrient enrichment, though cobalt partially alleviated these negative effects. Notably, cobalt enhanced Zn and Co uptake but slightly reduced Fe content. The findings indicate that partial substitution of K_2_SO_4_ with KCl can maintain satisfactory wheat productivity and grain quality, particularly when combined with cobalt application, whereas complete replacement with KCl is not recommended. Therefore, integrating cobalt supplementation with partial K_2_SO_4_ replacement represents a cost-effective and sustainable fertilization strategy for improving wheat production under arid and marginal soil conditions.

## Introduction

Globally, cereal crops serve as the primary staple food, providing over 50% of the total caloric intake for the human population. Among these, wheat (*Triticum aestivum* L.) plays a critical role as a major source of both calories and dietary protein for the world’s ever-increasing population^1^. In Egypt, wheat production falls short of meeting domestic consumption needs, highlighting the urgent necessity to boost wheat yields in order to bridge the gap between production and demand. Consequently, considerable efforts have been directed toward optimizing agricultural practices and identifying the most effective cultivation methods to maximize the productivity and quality of different wheat cultivars^2^. Nutrient management in commercial crop production, particularly the application of both macro- and micronutrients, is vital not only for achieving high yields but also for meeting quality standards required by markets^3^. However, conventional fertilization practices tend to emphasize macronutrient application via synthetic fertilizers, often neglecting the importance of micronutrients^4^. Recent studies have shed light on the mechanisms of micronutrient uptake and transport in plants, as well as their critical roles in various physiological functions^5^. Moreover, several reports have proposed targeted strategies to enhance micronutrient management, contributing to the advancement of sustainable and efficient crop production systems^6^.

Potassium is a vital macronutrient that influences a wide range of biochemical, phenological, and physiological processes in plants^7,8^. Its availability regulates key functions such as water uptake, nutrient translocation, enzyme activation, photosynthesis, protein synthesis, osmoregulation, energy transfer, and the regulation of stomatal opening^9,10^. As a central element in several metabolic pathways, potassium significantly impacts both crop yield and quality. Similar to nitrogen and phosphorus, potassium plays a crucial role in enhancing crop productivity. Numerous studies have demonstrated the direct and indirect effects of potassium on increasing wheat yield^11,12^. For instance, Sedri et al. ^13^ reported that potassium application improved growth, yield components, nutrient uptake, straw production, and grain yield in wheat. Conversely, insufficient potassium levels result in yield reduction, even when other nutrients are adequately supplied. Thornburg et al. ^14^ found that potassium deficiency markedly decreased the number of tillers, grain filling, and overall yield development in wheat. Moreover, potassium-deficient plants not only produce lower yields but also exhibit increased susceptibility to stresses such as salinity, drought, and diseases. Adequate potassium availability is therefore essential for achieving optimum wheat growth, yield, and grain quality. For most agricultural soils, exchangeable potassium concentrations below approximately 80–120 mg K kg^−1^ soil (ammonium acetate extractable) are generally considered deficient for wheat production, although the critical level varies with soil texture, mineralogy, and calibration procedures^15,16^. Maintaining soil K above this critical range is necessary to sustain high crop productivity and efficient nutrient use.

Cobalt (Co) is a naturally occurring trace element found in soils and the atmosphere in various chemical forms. Although it is not classified as an essential nutrient for higher plants, cobalt is widely recognized as a beneficial element because of its important roles in several physiological and biochemical processes^17^. Cobalt is a constituent of various enzymes and coenzymes and contributes to nitrogen metabolism, enzyme activation, chlorophyll synthesis, and plant tolerance to environmental stresses^18,19^.

The beneficial effects of cobalt are particularly evident in leguminous crops, where it is required for the activity of nitrogen-fixing enzymes and supports the growth of diazotrophic bacteria. These microorganisms are associated not only with legumes but also with the rhizosphere of several cereal crops, including barley, maize, rice, sugarcane, and wheat^18^. The influence of cobalt on plant growth and metabolism depends on its concentration, plant species, developmental stage, and rhizosphere conditions^19^. The concentration of total cobalt in agricultural soils commonly ranges from 1 to 40 mg kg^−1^, depending on parent material, soil type, and environmental conditions^20^. However, cobalt availability to plants is strongly influenced by soil properties, particularly pH, organic matter content, and redox conditions, with alkaline and calcareous soils generally exhibiting lower Co availability^21^. Consequently, cobalt deficiency is more likely to occur in such soils, potentially limiting plant growth and productivity. Therefore, supplemental cobalt application may improve crop performance under conditions where cobalt availability is restricted.

Several studies have demonstrated the positive effects of cobalt application on crop growth, productivity, and nutrient accumulation^22,23^. Gad et al. ^24^ reported that foliar application of cobalt at 10 mg L^−1^ combined with 75% of the recommended nitrogen fertilizer rate significantly improved all growth parameters of common bean compared with the full nitrogen rate applied without cobalt. Similarly, Kandil and El-Maghraby^25^ found that soil application of cobalt sulfate at 10 mg kg^−1^ significantly enhanced wheat growth and yield attributes, including plant height, leaf number, and shoot and root biomass. Furthermore, Sahay et al. ^26^ demonstrated that the combined application of cobalt and potassium improved growth, yield components, nutrient uptake, and protein content in lentil plants. These findings suggest that cobalt supplementation can enhance crop productivity and nutritional quality, particularly under conditions where micronutrient availability may limit plant growth.

This experiment aimed to evaluate the agronomic and nutritional impacts of substituting potassium sulfate with potassium chloride either fully or in varying proportions together with cobalt supplementation, to identify fertilizer strategies that optimize wheat growth, yield, and grain quality while reducing input costs. Specifically, the study assessed whether partial or complete replacement of the higher-cost potassium sulfate with the more economical potassium chloride could sustain crop performance, and how cobalt application modulates these outcomes under field conditions.

## Materials and methods

### Experimental field site and study objective

**Fig. 1:**
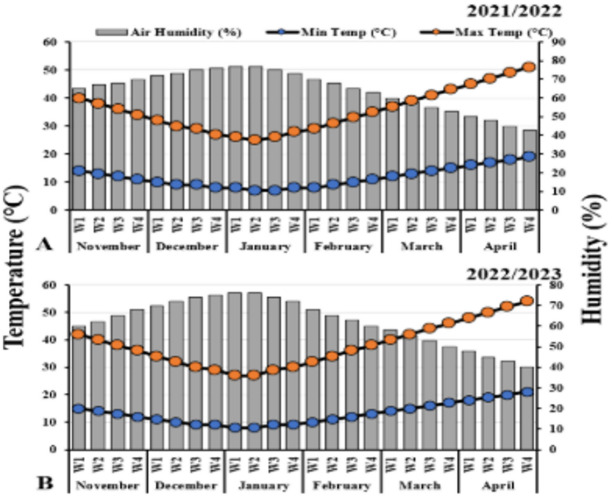
Weather of two growing seasons 2021/2022 (**A**) and 2022/2023 (**B**).

Field experiments were conducted at the Research and Production Station of the National Research Centre, located in El-Nubaryia, Behaira Governorate, in the Nile Delta region of Egypt (30°23′48′′ N, 30°18′59′′ E). Wheat (*Triticum aestivum* cv. Giza-95), kindly provided by the Wheat Research Section of the Field Crops Institute, ARC, Giza, was sown on November 16 and 19 during the 2021/2022 and 2022/2023 growing seasons, respectively. The experiment also aimed to determine whether partial substitution of K_2_SO_4_ with the less expensive KCl could maintain wheat productivity while reducing fertilizer costs. Weather data for the two growing seasons (2021/2022 and 2022/2023), obtained from the Egyptian Meteorological Authority (EMA, Cairo, Egypt; http://ema.gov.eg), are presented in Fig. 1. The study aimed to evaluate the role of cobalt in enhancing the efficiency of potassium fertilization in wheat cultivation.

### Soil analysis

Composite soil samples were taken from a depth of 0–30 cm, then air-dried, ground, and passed through a 2 mm sieve, following the method of Carter and Gregorich^27^. Soil texture was determined using the pipette method as outlined by Page et al.^29^. The calcium carbonate (CaCO_3_) content was analyzed using Jackson’s calcimeter method^28^. Organic matter in the soil was measured via dichromate oxidation, while calcium carbonate was again quantified with the calcimeter method of Jackson^28^. Soil salinity was evaluated by measuring the electrical conductivity (EC) of a 1:1 soil-to-water extract using an EC meter (Lovibond 200 Con, Germany). Soil pH was measured in a 1:2 soil-water suspension using a pH meter (Hanna Instruments pH 211, Romania)^28^. Available nitrogen (N) was extracted using 1% potassium sulfate (K_2_SO_4_) at a 1:5 soil-to-solution ratio, and 20 mL of this extract was distilled with 1 g of Devarda’s alloy using a micro-Kjeldahl apparatus^28^. Available phosphorus (P) was extracted with 0.5 M sodium bicarbonate (pH 8.5) and measured by the stannous chloride method; absorbance was read at 660 nm using a spectrophotometer (Unico 2000UV, Germany)^28^. Potassium (K) levels were determined with a flame photometer (Jenway 7PFP, England), as per Jackson’s protocol^28^. Micronutrients including iron (Fe), manganese (Mn), zinc (Zn), and copper (Cu) were assessed based on the methods described by Page^29^. In addition, soluble, available, and total cobalt (Co) concentrations in the soil were analyzed using the procedure established by Ryan et al. ^30^. The primary physical and chemical characteristics of the experimental soil, based on a representative sample taken before planting, are summarized in Table 1. The experiment was conducted on half of the land during the first season (2021/2022), while the remaining half was left uncultivated for use in the second season (2022/2023).

**Table 1:** Some physical and chemical properties of El-Nubaria soil.

Particle size distribution %				Soil moisture constant %			
Sand	Silt	Clay	Soil texture	Saturation	FC	WP	AW
Physical properties							
70.8	25.6	3.6	Sandy loam	32.0	19.2	6.1	13.1

				Soluble cations (meq^−1^L)				Soluble anions (meq^−1^L)			
pH 1:2.5	EC (dS m^−1^)	CaCO3%	OM %	Ca^++^	Mg^++^	K^+^	Na^+^	HCO_3_^−^	CO_3_	Cl^−^	SO_4_^−^
Chemical properties											
8.49	1.74	3.4	0.20	0.8	0.5	1.6	1.80	0.3	0.0	1.9	0.5

Cobalt			Total	Available		Available micronutriments			
ppm			mg 100 g^−1^ soil			ppm			
Soluble	Available	Total	N	P	K	Fe	Mn	Zn	Cu
0.35	4.88	9.88	15.1	13.3	4.49	4.46	2.71	4.52	5.2

FC, field capacity; WP, welting point; AW, available water.

### Experimental design and fertilizer treatments

The experimental layout followed a Randomized block split-split-plot design with three replicates. Years were allocated as main plot (2021/2022 and 2022/2023 growing seasons), and potassium fertilizer treatments were allocated to the sub-plots as follows: Control (100% recommended K_2_SO_4_), 75% K_2_SO_4_ + 25% KCl, 50% K_2_SO_4_ + 50% KCl, 25% K_2_SO_4_ + 75% KCl, and 100% KCl. Cobalt treatments were assigned to the sub-subplots: without cobalt and with cobalt (10 mg L^−1^). Each experimental plot measured 3.0 × 3.5 m (10.5 m^2^), with three replicate plots assigned to each treatment.

Potassium was supplied as potassium sulfate (K_2_SO_4_, 50% K_2_O and 18% S) and potassium chloride (KCl, 50% K_2_O) at the recommended rate of 238 kg ha^−1^ according to the treatment combinations. Cobalt was applied at a concentration of 10 mg L^−1^ because this level has previously been reported to enhance crop growth, yield, and nutrient uptake without causing phytotoxic effects^24,25^. Therefore, it was selected to evaluate its potential role in improving the efficiency of potassium fertilization in wheat under arid soil conditions. Cobalt was applied once at the seedling stage (third-leaf stage) through irrigation as cobalt sulfate (CoSO_4_·7 H_2_O, approximately 21% Co) at a concentration of 10 mg L^−1^.

Standard agricultural practices for wheat growth and production were followed in accordance with the recommendations of the Egyptian Ministry of Agriculture. A drip irrigation system was employed to ensure efficient water use. Irrigation hoses were spaced 30 cm apart, with drippers positioned at 30 cm intervals. Wheat was planted around the hoses, maintaining a spacing of 10 cm between planting holes. A fertilizer injector system was used to blend fertilizers with irrigation water, utilizing a vacuum generated by water flow to deliver nutrients directly to the root zone. Before sowing, 238 kg ha^−1^ of mono superphosphate (15.5% P_2_O_5_) was incorporated into the soil. Ammonium sulfate (20.5% N) was applied at 380.8 kg ha^−1^. Nitrogen fertilization continued until 60 days after planting, while potassium fertilization was supplied from 60 days after planting until spike emergence. Both nitrogen and potassium fertilizers were applied through the irrigation system to ensure uniform distribution and efficient uptake.

### Measurement of plant growth

Following the heading stage, wheat plant samples were randomly selected from each plot to assess vegetative growth characteristics. The recorded parameters included plant height (cm), days to 50% heading, number of tillers per square meter, and vegetative dry biomass (g plant^−1^). Plant height was measured from the soil surface to the tip of the tallest spike. Days to 50% heading were recorded as the number of days from sowing until approximately 50% of the plants in each plot had emerged spikes. The number of tillers was counted within a one-square-meter area in each plot and expressed as tillers m^−2^. Fresh biomass was measured immediately after sampling, and dry biomass was determined after oven-drying the samples at 70 °C for 72 h until constant weight. All measurements were performed according to the method described by Cotennie^31^.

### Measurement of grain yield

At the end of the growing season, ten tillers were randomly chosen from each plot to assess yield-related attributes. The evaluated parameters included spike length (cm), number of spikelets per spike, number of grains per spike, grain weight per spike (g), and the weight of 1,000 grains (g). All measurements were performed in accordance with the methods described by Gabal et al. ^32^.

### Measurement of nutritional status and chemical constituents

At harvest, wheat plant samples were collected from a one-square-meter area within each plot to evaluate the nutritional status and chemical composition of the grains. Analyses included the concentrations of macronutrients (nitrogen, phosphorus, and potassium), micronutrients (iron, manganese, zinc, and copper), as well as cobalt. Additionally, key chemical components such as total protein, total carbohydrates, and total soluble sugars (%) were measured. All analyses were conducted according to the standard procedures described by Cottenie et al. ^33^.

### Determination of chemical constituents

At harvest, mature wheat grain samples were collected from each experimental plot, air-dried, ground to pass through a fine sieve, and used for the determination of protein, total carbohydrates, starch, total soluble sugars, and mineral nutrient concentrations using standard analytical methods.

#### Total protein determination

Grain protein content was determined using the Kjeldahl method according to AOAC^34^. Total nitrogen was measured after acid digestion, distillation, and titration, and protein content was calculated by multiplying the nitrogen concentration by a conversion factor of 6.25.

#### Total carbohydrates determination

Total carbohydrate content was determined using the phenol-sulfuric acid method described by DuBois et al. ^35^. The absorbance was measured at 490 nm, and carbohydrate concentration was calculated using a glucose standard curve.

#### Starch content determination

Grain starch content was determined according to the method of McCready et al. ^36^. Starch was extracted by perchloric acid hydrolysis and quantified colorimetrically by measuring the starch-iodine complex at 620 nm. Results were expressed as a percentage of grain dry weight.

#### Total soluble sugars determination

Total soluble sugar content was determined using the anthrone method described by Ebell^37^. Soluble sugars were extracted with hot water and quantified colorimetrically at 620 nm using a glucose standard curve. Results were expressed as a percentage of grain dry weight.

### Statistical analysis

Data collected during the 2021/2022 and 2022/2023 growing seasons were subjected to a combined analysis using a three-way analysis of variance (ANOVA) in Statistix software (Version 8.1; Analytical Software, Tallahassee, FL, USA). The analysis considered year (Y), potassium source treatment (K), and cobalt application (Co) as fixed factors, and their interactions (Y × K, Y × Co, K × Co, and Y × K × Co) were tested. Prior to analysis, the assumptions of ANOVA, including normality of residuals and homogeneity of variance, were checked. When ANOVA indicated significant treatment effects (*P* ≤ 0.05), mean comparisons were performed using Tukey’s Honestly Significant Difference (HSD) test at the 5% probability level^38^. Main effects and interaction means were separated according to Tukey’s HSD procedure, and means followed by different letters were considered significantly different.

## Results

### Vegetative growth

The results presented in Table 2 demonstrate that potassium source, cobalt application, and their interaction significantly influenced wheat vegetative growth traits, including plant height and days to 50% heading. Plant height increased significantly with cobalt application across all potassium treatments, indicating that cobalt promoted vegetative growth regardless of the potassium source. On average, cobalt increased plant height by 6.2% compared with the untreated control. The greatest plant height was obtained with the 100% K_2_SO_4_ treatment combined with cobalt, whereas the shortest plants were recorded under the 100% KCl treatment without cobalt.

Potassium source also markedly affected vegetative growth. Plant height progressively declined as the proportion of KCl increased, with the following order: 100% K_2_SO_4_ > 75% K_2_SO_4_ + 25% KCl > 50% K_2_SO_4_ + 50% KCl > 25% K_2_SO_4_ + 75% KCl > 100% KCl. Nevertheless, all partial substitution treatments produced significantly greater plant height than complete replacement with KCl, indicating that partial substitution of K_2_SO_4_ maintained better vegetative performance.

A similar trend was observed for days to 50% heading. Plants receiving 100% K_2_SO_4_ exhibited the longest time to heading, whereas those fertilized solely with KCl reached heading earlier. Cobalt application slightly delayed heading (approximately 3.7%), particularly under the 100% K_2_SO_4_ and 75% K_2_SO_4_ + 25% KCl treatments, reflecting prolonged vegetative growth before reproductive development. Year-to-year variation was relatively small, with plants in the 2022/2023 season being slightly taller and reaching 50% heading marginally later than those grown in 2021/2022.

**Table 2:** Influence of cobalt and potassium sources on plant height and days to 50% heading in wheat.

Treatments		Plant height (cm)				No. of Days to 50% heading			
		21/22	22/23	Mean (K*Co)	Mean (Co)	21/22	22/23	Mean (K*Co)	Mean (Co)
Co(-)	100KS	117.81 f.	118.99 e	118.40 C	106.84 B	89.35 d	93.69 b	89.80 B	79.70 B
	75KS + 25KCl	117.78 f.	118.96 e	118.37 C		84.52 g	88.54 e	84.94 D	
	50KS + 50KCl	107.60 j	108.68 i	108.14 E		80.83 k	82.62 i	81.24 F	
	25KS + 75KCl	99.22 o	100.21 n	99.72 H		73.44 o	75.76 m	73.81 H	
	100KCl	89.12 q	90.01 p	89.57 I		68.38 s	70.55 q	68.72 J	
Co(+)	100KS	123.70 b	124.94 a	124.32 A	113.48 A	90.24 c	94.63 a	94.16 A	82.64A
	75KS + 25KCl	120.92 d	122.13 c	121.53 B		85.37 f.	89.43 d	88.98 C	
	50KS + 50KCl	110.84 h	111.95 g	111.40 D		81.64 j	83.45 h	83.04 E	
	25KS + 75KCl	106.21 k	107.27 j	106.74 F		74.17 n	76.52 l	76.14 G	
	100KCl	102.92 m	103.95 l	103.44 G		69.06 r	71.26 p	70.90 I	
Mean (Y)		109.61 B	110.71 A	-	-	80.77 B	81.58 A	-	-
LSD ≥ 5%	Y	0.11				0.19			
	K	0.25				0.19			
	Co	0.11				0.09			
	Y*K	0.41				0.32			
	Y*Co	0.21				0.16			
	K*Co	0.41				0.32			
	Y*K*Co	0.66				0.52			

Values within a column (Mean) followed by different letters are significantly different according to Tukey HSD test at *p* ≤ *0.05* during the 2021/2022 and 2022/2023 growing seasons*.* Where: 100KS = 100% K_2_ So_4_; 75KS + 25KCl** = **75% K_2_ So_4_ + 25% KCl; 50KS + 50KCl** = **50% K_2_ So_4_ + 50% KCl; 25KS + 75KCl = 25% K_2_ So_4_ + 75% KCl; 100KCl = 100% KCl; Co(-) = without cobalt; Co( +) = with Cobalt; Y = Years (Factor A); K = Source of Potassium nutrition (Factor B); Co (Factor C).

The results presented in Table 3 show that potassium source, cobalt application, and their interaction significantly affected the number of tillers per m^2^ and vegetative dry matter of wheat plants. Cobalt application significantly enhanced both traits, increasing the number of tillers by approximately 1.9% and vegetative dry matter by 8.2% compared with the untreated control, indicating its positive role in promoting vegetative growth.

Potassium source also had a marked influence on vegetative growth. The highest number of tillers and greatest vegetative dry matter were obtained with the 100% K_2_SO_4_ treatment, whereas the 100% KCl treatment produced the lowest values. As the proportion of KCl increased, both tiller number and vegetative dry matter progressively declined. Nevertheless, all partial replacement treatments (75% K_2_SO_4_ + 25% KCl, 50% K_2_SO_4_ + 50% KCl, and 25% K_2_SO_4_ + 75% KCl) performed significantly better than complete replacement with KCl, indicating that partial substitution of K_2_SO_4_ maintained superior vegetative growth.

Seasonal variation was relatively small, with the 2022/2023 growing season producing slightly higher tiller numbers and vegetative dry matter than the 2021/2022 season. Although the magnitude of the responses varied between seasons, the overall trend remained consistent, with cobalt application enhancing vegetative growth and K_2_SO_4_ outperforming KCl in supporting tillering and biomass accumulation.

**Table 3:** Influence of cobalt and potassium sources on no. of tillers per m^2^ and weeds dry weight in wheat plants.

Treatments		No. of Tillers/ m^2^				Weeds dry weight g/ plant			
		21/22	22/23	Mean (K*Co)	Mean (Co)	21/22	22/23	Mean (K*Co)	Mean (Co)
Co(-)	100KS	393.70 e	397.64 d	395.67 C	381.12 B	80.11 e	80.91 d	80.51 C	68.90 B
	75KS + 25KCl	387.82 h	391.70 f.	389.76 D		75.62 g	76.377 f.	76.00 D	
	50KS + 50KCl	379.94 l	383.74 j	381.84 F		68.24 j	68.920 i	68.58 F	
	25KS + 75KCl	371.87 o	375.59 n	373.73 H		61.92 o	62.54 n	62.23 I	
	100KCl	362.79 r	366.42 q	364.61 J		56.88 q	57.45 p	57.17 J	
Co( +)	100KS	404.08 b	408.12 a	406.10 A	388.37 A	84.83 b	85.68 a	85.25 A	74.55 A
	75KS + 25KCl	398.03 d	402.01 c	400.02 B		80.69 d	81.50 c	81.09 B	
	50KS + 50KCl	386.11 i	389.97 g	388.04 E		74.94h	75.69 g	75.32 E	
	25KS + 75KCl	377.40 m	381.17 k	379.29 G		67.22 k	67.89 j	67.56 G	
	100KCl	366.59 q	370.26 p	368.42 I		63.19 m	63.82 l	63.51 H	
Mean (Y)		382.83 B	386.66 A	-	-	71.364 B	72.078 A	-	-
LSD ≥ 5%	Y	0.14				0.09			
	K	0.31				0.21			
	Co	0.14				0.09			
	Y*K	0.52				0.35			
	Y*Co	0.26				0.18			
	K*Co	0.52				0.35			
	Y*K*Co	0.83				0.56			

Values within a column (Mean) followed by different letters are significantly different according to Tukey HSD test at *p* ≤ *0.05* during the 2021/2022 and 2022/2023 growing seasons*.* Where: 100KS = 100% K_2_ So_4_; 75KS + 25KCl** = **75% K_2_ So_4_ + 25% KCl; 50KS + 50KCl** = **50% K_2_ So_4_ + 50% KCl; 25KS + 75KCl = 25% K_2_ So_4_ + 75% KCl; 100KCl = 100% KCl; Co(-) = without cobalt; Co( +) = with Cobalt; Y = Years (Factor A); K = Source of Potassium nutrition (Factor B); Co (Factor C).

### Yield characteristics

Table 4; Fig. 1 demonstrates that potassium source and cobalt application significantly influenced all yield components and total grain yield of wheat. Application of cobalt significantly affected both the number of spikes per m^2^ and spike length in wheat plants (Table 4). On average, cobalt increased the number of spikes from 377.59 spikes/m^2^ without cobalt to 391.09 spikes/m^2^ with cobalt, showing its positive influence on spike formation. Likewise, spike length increased from 10.35 cm without cobalt to 15.94 cm with cobalt, indicating that cobalt had a strong promotive effect on spike elongation. Potassium source also had a marked impact. The highest spike density was achieved with 100% K_2_SO_4_ (100KS) (390.36 spikes/m^2^ without cobalt and 408.29 spikes/m^2^ with cobalt), while the lowest was observed with 100% KCl (100KCl) (364.14 spikes/m^2^ without cobalt and 373.09 spikes/m^2^ with cobalt). Mixed sources produced intermediate values. A similar trend was seen for spike length: the longest spikes were recorded under 100KS with cobalt (16.71 cm), whereas the shortest were obtained with 100KCl without cobalt (9.66 cm). All mixing ratios of potassium sulfate and potassium chloride produced better results than potassium chloride alone. Seasonal variation was also evident. The 2022/23 season produced slightly higher values (386.25 spikes/m^2^ and 13.21 cm spike length) compared with 2021/22 (382.42 spikes/m^2^ and 13.08 cm).

**Table 4:** Influence of cobalt and potassium sources on no. of spike per m^2^ and spike length in wheat plants.

Treatments		No. of Spike/ m^2^				Spike length (cm)			
		21/22	22/23	Mean (K*Co)	Mean (Co)	21/22	22/23	Mean (K*Co)	Mean (Co)
Co(-)	100KS	388.42 h	392.30 f.	390.36 D	377.59 B	10.92 gh	11.03g	10.98 F	10.35 B
	75KS + 25KCl	380.71 k	384.52 i	382.62 E		10.74 h	10.85 h	10.79 G	
	50KS + 50KCl	377.87 l	381.65 j	379.76 G		10.26 i	10.36 i	10.31 H	
	25KS + 75KCl	369.21 p	372.90 n	371.06 I		9.90 jk	10.00 j	9.95 I	
	100KCl	362.33 r	365.95 q	364.14 J		9.66 l	9.76 kl	9.71 J	
Co( +)	100KS	406.26 b	410.32 a	408.29 A	391.09 A	16.56 a	16.73 a	16.64 A	15.94 A
	75KS + 25KCl	398.40 d	402.38 c	400.39 B		16.20 bc	16.36 b	16.28 B	
	50KS + 50KCl	389.72 g	393.62 e	391.67 C		15.96 d	16.12 cd	16.04 C	
	25KS + 75KCl	380.09 k	383.89 i	381.99 F		15.54 e	15.70 e	15.62 D	
	100KCl	371.23 o	374.94m	373.09 H		15.06 f.	15.21 f.	15.14 E	
Mean (Y)		382.42 B	386.25 A	-	-	13.08 B	13.21 A	-	-
LSD ≥ 5%	Y	0.13				0.03			
	K	0.31				0.07			
	Co	0.13				0.03			
	Y*K	0.51				0.11			
	Y*Co	0.26				0.06			
	K*Co	0.51				0.11			
	Y*K*Co	0.83				0.18			

Values within a column (Mean) followed by different letters are significantly different according to Tukey HSD test at *p* ≤ *0.05* during the 2021/2022 and 2022/2023 growing seasons*.* Where: 100KS = 100% K_2_ So_4_; 75KS + 25KCl** = **75% K_2_ So_4_ + 25% KCl; 50KS + 50KCl** = **50% K_2_ So_4_ + 50% KCl; 25KS + 75KCl = 25% K_2_ So_4_ + 75% KCl; 100KCl = 100% KCl; Co(-) = without cobalt; Co( +) = with Cobalt; Y = Years (Factor A); K = Source of Potassium nutrition (Factor B);Co (Factor C).

Application of cobalt significantly improved both spike grain weight and 1000-grain weight in wheat (Table 5). On average, cobalt increased spike grain weight from 1.73 g without cobalt to 1.80 g with cobalt, and 1000-grain weight from 36.42 g to 39.25 g, confirming its strong role in enhancing grain filling and kernel development. Potassium source also showed a clear effect: 100% K_2_SO_4_ (100KS) recorded the highest values (1.79 g spike grain weight without cobalt and 1.81 g with cobalt; 37.90 g and 42.03 g for 1000-grain weight, respectively), while 100% KCl (100KCl) produced the lowest (1.65 g spike grain weight without cobalt and 1.79 g with cobalt; 35.27 g and 37.65 g for 1000-grain weight, respectively), with mixed sources yielding intermediate results. All mixing ratios of potassium sulfate and potassium chloride produced better results than potassium chloride alone. Seasonal differences were minor, but slightly higher averages were obtained in 2022/23 (1.77 g spike grain weight and 38.02 g 1000-grain weight) compared to 2021/22 (1.76 g and 37.65 g, respectively).

**Table 5:** Influence of cobalt and potassium sources on spike grain weight and 1000 grains weight in wheat plants.

Treatments		Spike grain weight (g)				1000 grains weight (g)			
		21/22	22/23	Mean (K*Co)	Mean (Co)	21/22	22/23	Mean (K*Co)	Mean (Co)
Co(-)	100KS	1.78 ef	1.80 cd	1.79 C	1.73 B	37.80 fg	38.18 e	37.99 C	36.42 B
	75KS + 25KCl	1.78 f.	1.80 d	1.79 C		36.69 j	37.06 i	36.88 E	
	50KS + 50KCl	1.74 h	1.76 g	1.75 D		36.19 l	36.55 k	36.37 F	
	25KS + 75KCl	1.67 j	1.69 i	1.68 E		35.40 n	35.75 m	35.58 G	
	100KCl	1.64 l	1.66 k	1.65 F		35.09 o	35.44 n	35.27 H	
Co( +)	100KS	1.80bcd	1.82 a	1.81 A	1.80 A	41.82 b	42.24 a	42.03 A	39.25 A
	75KS + 25KCl	1.80 d	1.81 ab	1.81 B		40.75 d	41.16 c	40.96 B	
	50KS + 50KCl	1.79 de	1.81 bc	1.80 B		37.72 g	38.10 e	37.91 C	
	25KS + 75KCl	1.78 ef	1.80bcd	1.79 C		37.53 h	37.91 f.	37.72 D	
	100KCl	1.78 f.	1.80 d	1.79 C		37.46 h	37.83 fg	37.65 D	
Mean (Y)		1.76 B	1.77 A	-	-	37.65 B	38.02 A	-	-
LSD ≥ 5%	Y	0.001				0.02			
	K	0.004				0.05			
	Co	0.001				0.02			
	Y*K	0.006				0.08			
	Y*Co	0.003				0.04			
	K*Co	0.006				0.08			
	Y*K*Co	0.01				0.134			

Values within a column (Mean) followed by different letters are significantly different according to Tukey HSD test at *p* ≤ *0.05* during the 2021/2022 and 2022/2023 growing seasons*.* Where: 100KS = 100% K_2_ So_4_; 75KS + 25KCl** = **75% K_2_ So_4_ + 25% KCl; 50KS + 50KCl** = **50% K_2_ So_4_ + 50% KCl; 25KS + 75KCl = 25% K_2_ So_4_ + 75% KCl; 100KCl = 100% KCl; Co(-) = without cobalt; Co( +) = with Cobalt; Y = Years (Factor A); K = Source of Potassium nutrition (Factor B); Co (Factor C).

Application of cobalt significantly enhanced both straw yield and grain yield in wheat (Table 6). On average, straw yield increased from 4.12 ton/ha without cobalt to 4.62 ton/ha with cobalt, while grain yield rose from 8.89 ton/ha to 9.13 ton/ha, confirming cobalt’s beneficial role in improving overall biomass and productivity. Potassium source also had a marked effect, with 100% K_2_SO_4_ (100KS) producing the highest yields (4.19 ton/ha straw and 9.18 ton/ha grain without cobalt, and 4.80 and 9.35 ton/ha with cobalt), whereas 100% KCl (100KCl) resulted in the lowest values (3.99 and 8.40 ton/ha without cobalt, and 4.25 and 8.96 ton/ha with cobalt). Mixed potassium sources gave intermediate results. All mixing ratios of potassium sulfate and potassium chloride produced better results than potassium chloride alone. Seasonal effects were also noted, with slightly higher means in 2022/23 (4.39 ton/ha straw and 9.06 ton/ha grain) compared to 2021/22 (4.34 ton/ha and 8.97 ton/ha, respectively).

**Table 6:** Influence of cobalt and potassium sources on straw yield ton per hectare and grain yield ton per hectare in wheat plants.

Treatments		Straw Yield ton/ha^-1^				Grain Yield ton/ha^-1^			
		21/22	22/23	Mean (K*Co)	Mean (Co)	21/22	22/23	Mean (K*Co)	Mean (Co)
Co(-)	100KS	4.17 k	4.21 ij	4.19 F	4.12 B	9.13 d	9.22 c	9.18 C	8.89 B
	75KS + 25KCl	4.15 k	4.19 j	4.17 G		9.03 f.	9.12 d	9.08 D	
	50KS + 50KCl	4.11 l	4.15 k	4.13 H		8.98 h	9.07 e	9.03 E	
	25KS + 75KCl	4.08 m	4.12 l	4.10 I		8.73 k	8.82 j	8.77 G	
	100KCl	3.97 o	4.01 n	3.99 J		8.36 m	8.44 l	8.40 H	
Co( +)	100KS	4.78 b	4.83 a	4.80 A	4.62 A	9.30 b	9.39 a	9.35 A	9.13 A
	75KS + 25KCl	4.70 d	4.75 c	4.72 B		9.22 c	9.31 b	9.27 B	
	50KS + 50KCl	4.66 e	4.71 d	4.68 C		9.04 f.	9.13 d	9.09 D	
	25KS + 75KCl	4.59 g	4.64 f.	4.61 D		8.97 h	9.06 e	9.02 E	
	100KCl	4.23 i	4.27 h	4.25 E		8.91 i	9.00 g	8.96 F	
Mean (Y)		4.34 B	4.39 A	-	-	8.97 B	9.06 A	-	-
LSD ≥ 5%	Y	0.003				0.003			
	K	0.01				0.01			
	Co	0.004				0.003			
	Y*K	0.014				0.01			
	Y*Co	0.01				0.005			
	K*Co	0.01				0.01			
	Y*K*Co	0.02				0.016			

Values within a column (Mean) followed by different letters are significantly different according to Tukey HSD test at *p* ≤ *0.05* during the 2021/2022 and 2022/2023 growing seasons*.* Where: 100KS = 100% K_2_ So_4_; 75KS + 25KCl** = **75% K_2_ So_4_ + 25% KCl; 50KS + 50KCl** = **50% K_2_ So_4_ + 50% KCl; 25KS + 75KCl = 25% K_2_ So_4_ + 75% KCl; 100KCl = 100% KCl; Co(-) = without cobalt; Co( +) = with Cobalt; Y = Years (Factor A); K = Source of Potassium nutrition (Factor B); Co (Factor C).

### Nutritional status

Application of cobalt significantly improved the nutritional status of wheat grains in terms of nitrogen (N), phosphorus (P), and potassium (K) concentrations (Table 7). On average, cobalt increased grain N from 1.38% without cobalt to 1.40% with cobalt, P from 0.87% to 0.88% (a slight improvement), and K from 2.80 to 2.93%, indicating its positive effect on nutrient accumulation. Potassium source also played an important role: 100% K_2_SO_4_ (100KS) consistently produced the highest nutrient contents (1.44% and 1.49% N; 0.88% and 0.89% P; 2.89% and 3.06% K without and with cobalt, respectively), whereas 100% KCl (100KCl) recorded the lowest values (1.32%–1.32% N, 0.86%–0.86% P, and 2.69%–2.78% Kwithout and with cobalt, respectively). Mixed potassium sources showed intermediate performance. All mixing ratios of potassium sulfate and potassium chloride produced better results than potassium chloride alone. Seasonal variation was minor, with 2022/23 generally recording slightly higher nutrient values (1.40% N, 0.88% P, and 2.88% K) compared to 2021/22 (1.39% N, 0.87% P, and 2.85% K).

**Table 7:** Influence of cobalt and potassium sources on grain nutritional status (N, P, and K) in wheat plants.

Treatments		N (%)				P (%)				K (%)			
		21/22	22/23	Mean (K*Co)	Mean (Co)	21/22	22/23	Mean (K*Co)	Mean (Co)	21/22	22/23	Mean (K*Co)	Mean (Co)
Co(-)	100KS	1.43 e	1.44 d	1.44 C	1.38 B	0.87cde	0.88 bc	0.88 BC	0.87 B	2.88 g	2.91 f.	2.89 D	2.80 B
	75KS + 25KCl	1.41 f.	1.42 e	1.42 D		0.87 de	0.88 bc	0.88 BCD		2.86 h	2.88 g	2.87 E	
	50KS + 50KCl	1.37 h	1.38 g	1.38 F		0.87 ef	0.87 cde	0.87 DE		2.79 j	2.82 i	2.80 G	
	25KS + 75KCl	1.34 j	1.35 i	1.35 H		0.86 f.	0.87ef	0.86 F		2.73 m	2.76 l	2.75 I	
	100KCl	1.31 l	1.32 k	1.32 I		0.85 g	0.86 f.	0.86 FG		2.68 o	2.71 n	2.69 J	
Co( +)	100KS	1.48 b	1.49 a	1.49 A	1.40 A	0.88 bc	0.89 a	0.89 A	0.88 A	3.04 b	3.07 a	3.06 A	2.93 A
	75KS + 25KCl	1.46 c	1.47 b	1.47 B		0.88bcd	0.88 ab	0.88 AB		3.02 c	3.05 b	3.04 B	
	50KS + 50KCl	1.39 g	1.40 f.	1.40 E		0.87 ef	0.88bcd	0.87 CD		2.94 e	2.97 d	2.96 C	
	25KS + 75KCl	1.35 i	1.36 h	1.36 G		0.86 f.	0.87 de	0.87 EF		2.82 i	2.85 h	2.84F	
	100KCl	1.31 l	1.32 k	1.32 I		0.86 f.	0.87ef	0.86 F		2.77 k	2.80 j	2.78 H	
Mean (Co)		1.39 B	1.40 A	-	-	0.87 B	0.88A	-	-	2.85 B	2.88 A	-	-
LSD ≥ 5%	Y	0.002				0.004				0.002			
	K	0.003				0.004				0.004			
	Co	0.001				0.002				0.002			
	Y*K	0.005				0.006				0.006			
	Y*Co	0.002				0.003				0.003			
	K*Co	0.005				0.006				0.006			
	Y*K*Co	0.009				0.009				0.01			

Values within a column (Mean) followed by different letters are significantly different according to Tukey HSD test at *p* ≤ *0.05* during the 2021/2022 and 2022/2023 growing seasons*.* Where: 100KS = 100% K_2_ So_4_; 75KS + 25KCl** = **75% K_2_ So_4_ + 25% KCl; 50KS + 50KCl** = **50% K_2_ So_4_ + 50% KCl; 25KS + 75KCl = 25% K_2_ So_4_ + 75% KCl; 100KCl = 100% KCl; Co(-) = without cobalt; Co( +) = with Cobalt; Y = Years (Factor A); K = Source of Potassium nutrition (Factor B); Co (Factor C).

Application of cobalt significantly improved the grain nutritional status of wheat in terms of micronutrients Mn, Zn, and Cu (Table 8). On average, cobalt increased Mn from 34.43 mg/L without cobalt to 35.62 mg/L with cobalt, Zn from 20.46 to 21.99 mg/L, and Cu from 15.90 mg/L to 17.33 mg/L, highlighting its positive influence on micronutrient accumulation in grains. Potassium source also had a clear effect, with 100% K_2_SO_4_ (100KS) producing the highest concentrations (Mn: 37.69–38.19 mg/L; Zn: 22.51–24.72 mg/L; Cu: 18.59–20.40 mg/L without and with cobalt, respectively), while 100% KCl (100KCl) gave the lowest values (Mn: 31.05–32.76 mg/L; Zn: 17.99–19.20 mg/L; Cu: 13.07–14.27 mg/L without and with cobalt, respectively). All mixing ratios of potassium sulfate and potassium chloride produced better results than potassium chloride alone. Seasonal variation was minimal, with slightly higher average values in 2022/23 (Mn: 35.20 mg/L, Zn: 21.33 mg/L, Cu: 16.69 mg/L) compared to 2021/22 (Mn: 34.85 mg/L, Zn: 21.12 mg/L, Cu: 16.53 mg/L).

**Table 8:** Influence of cobalt and potassium sources on grain nutritional status (Mn, Zn, and Cu) in wheat plants.

Treatments		Mn (mg/L)				Zn (mg/L)				Cu (mg/L)			
		21/22	22/23	Mean (K*Co)	Mean (Co)	21/22	22/23	Mean (K*Co)	Mean (Co)	21/22	22/23	Mean (K*Co)	Mean (Co)
Co(-)	100KS	37.50 e	37.88 c	37.69 C	34.43 B	22.40 f.	22.62 e	22.51 C	20.46 B	18.50 f.	18.69 e	18.59 C	15.90 B
	75KS + 25KCl	37.20 f.	37.57 de	37.39 D		22.20 g	22.42 f.	22.31 D		18.30 g	18.48 f.	18.39 D	
	50KS + 50KCl	34.10 j	34.44 i	34.27 F		20.70 j	20.91 i	20.81 F		15.80 j	15.96 j	15.88 F	
	25KS + 75KCl	31.60 o	31.92 n	31.76 I		18.60 p	18.79 o	18.69 I		13.50 m	13.64 m	13.57 I	
	100KCl	30.90 q	31.21 p	31.05 J		17.90 r	18.08 q	17.99 J		13.00 n	13.13 n	13.07 J	
Co( +)	100KS	38.00 bc	38.38 a	38.19 A	35.62 A	24.60 b	24.85 a	24.72 A	21.99 A	20.30 b	20.50 a	20.40 A	17.33 A
	75KS + 25KCl	37.70 d	38.08 b	37.89 B		23.90 d	24.14 c	24.02 B		19.80 d	20.00 c	19.90 B	
	50KS + 50KCl	36.00 h	36.36 g	36.18 E		22.00 h	22.22 g	22.11 E		17.20 i	17.37 h	17.28 E	
	25KS + 75KCl	32.90 l	33.23 k	33.06 G		19.80 l	20.00 k	19.90 G		14.70 k	14.85 k	14.77 G	
	100KCl	32.60 m	32.93 l	32.76 H		19.10 n	19.29 m	19.20 H		14.20 l	14.34 l	14.27 H	
Mean (Co)		34.85 B	35.20 A	-	-	21.12 B	21.33 A	-	-	16.53 B	16.69 A	-	-
LSD ≥ 5%	Y	0.03				0.02				0.027			
	K	0.06				0.05				0.06			
	Co	0.02				0.02				0.028			
	Y*K	0.10				0.09				0.10			
	Y*Co	0.05				0.04				0.05			
	K*Co	0.10				0.08				0.10			
	Y*K*Co	0.17				0.14				0.17			

Values within a column (Mean) followed by different letters are significantly different according to Tukey HSD test at *p* ≤ *0.05* during the 2021/2022 and 2022/2023 growing seasons*.* Where: 100KS = 100% K_2_ So_4_; 75KS + 25KCl** = **75% K_2_ So_4_ + 25% KCl; 50KS + 50KCl** = **50% K_2_ So_4_ + 50% KCl; 25KS + 75KCl = 25% K_2_ So_4_ + 75% KCl; 100KCl = 100% KCl; Co(-) = without cobalt; Co( +) = with Cobalt; Y = Years (Factor A); K = Source of Potassium nutrition (Factor B);Co (Factor C).

Application of cobalt significantly influenced the nutritional status of wheat grains in terms of iron (Fe) and cobalt (Co) concentrations (Table 9). On average, cobalt slightly decreased Fe content from 228.94 mg/L without cobalt to 220.70 mg/L with cobalt, while it markedly increased grain Co content from 0.83 mg/L to 3.80 mg/L, reflecting its direct contribution to Co enrichment in grains. Potassium source also affected mineral accumulation: 100% K_2_SO_4_ (100KS) consistently produced the highest Fe and Co concentrations (236.18 mg/L Fe and 0.85 mg/L Co without cobalt; 228.13 mg/L Fe and 3.95 mg/L Co with cobalt), whereas 100% KCl (100KCl) recorded the lowest values (220.10 mg/L Fe and 0.82 mg/L Co without cobalt; 212.06 mg/L Fe and 3.63 mg/L Co with cobalt). All mixing ratios of potassium sulfate and potassium chloride produced better results than potassium chloride alone. Seasonal variation was minor, with 2022/23 showing slightly higher Fe (225.94 mg/L) and Co (2.33 mg/L) compared to 2021/22 (223.70 mg/L Fe and 2.30 mg/L Co). Overall, cobalt application enriched wheat grains with Co but reduced Fe concentration, and K_2_SO_4_ proved more effective than KCl in supporting higher micronutrient levels.

**Table 9:** Influence of cobalt and potassium sources on grain nutritional status (Fe and Co) in wheat plants.

Treatments		Fe (mg/L)				Cobalt (mg/L)			
		21/22	22/23	Mean (K*Co)	Mean (Co)	21/22	22/23	Mean (K*Co)	Mean (Co)
Co(-)	100KS	235.0 b	237.4 a	236.18 A	228.94 A	0.84 e	0.85 e	0.85 D	0.83 B
	75KS + 25KCl	233.0 c	235.3 b	234.17 B		0.82 e	0.83 e	0.82 D	
	50KS + 50KCl	228.0 f.	230.3 d	229.14 C		0.82 e	0.83 e	0.82 D	
	25KS + 75KCl	224.0 j	226.2 h	225.12 F		0.82 e	0.83e	0.82 D	
	100KCl	219.0 n	221.2 l	220.10 H		0.82 e	0.83e	0.82 D	
Co( +)	100KS	227.0 g	229.3 e	228.13 D	220.70 B	3.93 a	3.97 a	3.95 A	3.80 A
	75KS + 25KCl	225.0 i	227.3 g	226.13 E		3.93 a	3.97 a	3.95 A	
	50KS + 50KCl	220.0 m	222.2 k	221.10 G		3.78 bc	3.82 b	3.80 B	
	25KS + 75KCl	215.0 p	217.2 o	216.08 I		3.66 d	3.70 cd	3.68C	
	100KCl	211.0 r	213.1 q	212.06 J		3.61 d	3.65 d	3.63 C	
Mean (Y)		223.70 B	225.94 A	–	–	2.30 B	2.33 A	–	–
LSD ≥ 5%	Y	0.08				0.016			
	K	0.18				0.035			
	Co	0.07				0.015			
	Y*K	0.29				0.06			
	Y*Co	0.15				0.03			
	K*Co	0.29				0.05			
	Y*K*Co	0.47				0.09			

Values within a column (Mean) followed by different letters are significantly different according to Tukey HSD test at *p* ≤ *0.05* during the 2021/2022 and 2022/2023 growing seasons*.* Where: 100KS = 100% K_2_ So_4_; 75KS + 25KCl** = **75% K_2_ So_4_ + 25% KCl; 50KS + 50KCl** = **50% K_2_ So_4_ + 50% KCl; 25KS + 75KCl = 25% K_2_ So_4_ + 75% KCl; 100KCl = 100% KCl; Co(-) = without cobalt; Co( +) = with Cobalt; Y = Years (Factor A); K = Source of Potassium nutrition (Factor B);Co (Factor C).

### Chemical constituents

Application of cobalt significantly improved the chemical composition of wheat grains, particularly protein and carbohydrate contents (Table 10). On average, cobalt increased total protein concentration from 8.65% without cobalt to 8.83% with cobalt, while total carbohydrates also increased from 46.89% to 48.02%, confirming its positive role in enhancing grain nutritional quality. Potassium source also had a strong effect: 100% K_2_SO_4_ (100KS) produced the highest values for both protein (8.99% without cobalt and 9.30% with cobalt) and carbohydrates (50.50% and 51.46%, respectively), while 100% KCl (100KCl) recorded the lowest (8.39–8.44% protein and 44.42–45.43% carbohydrates). All mixing ratios of potassium sulfate and potassium chloride produced better results than potassium chloride alone. Seasonal variation was also evident, with slightly higher averages for protein and carbohydrate contents in 2022/23 (8.78% and 47.69%, respectively) compared to 2021/22 (8.69% and 47.22%, respectively).

**Table 10:** Influence of cobalt and potassium sources on chemical constituents of wheat grain (total proteins and total carbohydrates).

Treatments		Total proteins (%)				Total carbohydrates			
		21/22	22/23	Mean (K*Co)	Mean (Co)	21/22	22/23	Mean (K*Co)	Mean (Co)
Co(-)	100KS	8.940 f.	9.03 e	8.99 C	8.65 B	50.00 e	50.50 c	50.25 B	46.89 B
	75KS + 25KCl	8.81 h	8.90 g	8.86 D		48.20 h	48.68 g	48.44 D	
	50KS + 50KCl	8.56 l	8.65 k	8.60 F		46.10 l	46.56 k	46.33 F	
	25KS + 75KCl	8.38 r	8.46 o	8.42 I		44.80 o	45.25 n	45.02 I	
	100KCl	8.35 s	8.43 p	8.39 J		44.20 q	44.64 p	44.42 J	
Co( +)	100KS	9.25 b	9.34 a	9.30 A	8.83 A	51.20 b	51.71 a	51.46 A	48.02 A
	75KS + 25KCl	9.13 d	9.22 c	9.18 B		49.80 f.	50.30 d	50.05 C	
	50KS + 50KCl	8.69 j	8.78 i	8.73 E		47.00 j	47.47 i	47.24 E	
	25KS + 75KCl	8.44 p	8.52 m	8.48 G		45.70 m	46.16 l	45.93 G	
	100KCl	8.40 q	8.48 n	8.44 H		45.20 n	45.65 m	45.43 H	
Mean (Y)		8.69 B	8.78 A	–	–	47.22 B	47.69 A	–	–
LSD ≥ 5%	Y	0.003				0.025			
	K	0.007				0.06			
	Co	0.003				0.025			
	Y*K	0.012				0.09			
	Y*Co	0.006				0.05			
	K*Co	0.012				0.09			
	Y*K*Co	0.019				0.15			

Values within a column (Mean) followed by different letters are significantly different according to Tukey HSD test at *p* ≤ *0.05* during the 2021/2022 and 2022/2023 growing seasons*.* Where: 100KS = 100% K_2_ So_4_; 75KS + 25KCl** = **75% K_2_ So_4_ + 25% KCl; 50KS + 50KCl** = **50% K_2_ So_4_ + 50% KCl; 25KS + 75KCl = 25% K_2_ So_4_ + 75% KCl; 100KCl = 100% KCl; Co(-) = without cobalt; Co( +) = with Cobalt; Y = Years (Factor A); K = Source of Potassium nutrition (Factor B); Co (Factor C).

Cobalt application enhanced wheat grain biochemical constituents, particularly starch and total soluble sugars, across the two seasons (Table 11). On average, cobalt increased starch from 11.66% without cobalt to 12.06% with cobalt, and total soluble sugars from 5.22% to 5.47%, confirming its positive role in carbohydrate metabolism. Among potassium sources, 100% K_2_SO_4_ (100KS) produced the highest starch (12.36% without cobalt and 12.87% with cobalt) and sugars (5.80% and 6.09%, respectively), while 100% KCl (100KCl) consistently recorded the lowest values (10.86–11.36% starch and 4.78–4.81% sugars). Intermediate values were obtained with mixed K_2_SO_4_ + KCl treatments. All mixing ratios of potassium sulfate and potassium chloride produced better results than potassium chloride alone. Seasonal variation showed slightly higher averages in 2022/23 (11.92% starch, 5.37% sugars) compared to 2021/22 (11.88% starch, 5.32% sugars).

**Table 11:** Influence of cobalt and potassium sources on chemical constituents of wheat grain (starch and total soluble sugars).

Treatments		Total soluble sugars				Total Soluble sugars			
		21/22	22/23	Mean (K*Co)	Mean (Co)	21/22	22/23	Mean (K*Co)	Mean (Co)
Co(-)	100KS	12.30 d	12.80 b	12.36 B	11.66 B	5.79 ef	5.85 cd	5.82 BC	5.22 B
	75KS + 25KCl	12.10 f.	12.30 d	12.16 C		5.77 f.	5.83 de	5.80 C	
	50KS + 50KCl	11.60 k	11.90 h	11.66 F		4.86 ij	4.91 gh	4.89 E	
	25KS + 75KCl	11.20 n	11.70 j	11.26 H		4.81 kl	4.86 ij	4.84 F	
	100KCl	10.80 p	11.30 m	10.86 I		4.76 m	4.81 kl	4.78 G	
Co( +)	100KS	12.42 c	12.93 a	12.87 A	12.06 A	6.85 b	6.92 a	6.89 A	5.47 A
	75KS + 25KCl	12.22 e	12.42 c	12.36 B		5.81 def	5.87 c	5.84 B	
	50KS + 50KCl	11.72 j	12.02 g	11.96 D		4.90 hi	4.95 g	4.93 D	
	25KS + 75KCl	11.31 m	11.82 i	11.76 E		4.84 jk	4.89 hi	4.87 E	
	100KCl	10.91 o	11.41 l	11.36 G		4.79 lm	4.84 jk	4.81 F	
Mean (Y)		11.88 B	11.92 A	-	–	5.32 B	5.37 A	–	–
LSD ≥ 5%	Y	0.006				0.007			
	K	0.015				0.016			
	Co	0.006				0.007			
	Y*K	0.025				0.027			
	Y*Co	0.012				0.014			
	K*Co	0.024				0.027			
	Y*K*Co	0.04				0.04			

Values within a column (Mean) followed by different letters are significantly different according to Tukey HSD test at *p* ≤ *0.05* during the 2021/2022 and 2022/2023 growing seasons*.* Where: 100KS = 100% K_2_ So_4_; 75KS + 25KCl** = **75% K_2_ So_4_ + 25% KCl; 50KS + 50KCl** = **50% K_2_ So_4_ + 50% KCl; 25KS + 75KCl = 25% K_2_ So_4_ + 75% KCl; 100KCl = 100% KCl; Co(-) = without cobalt; Co( +) = with Cobalt; Y = Years (Factor A); K = Source of Potassium nutrition (Factor B); Co (Factor C).

### Regression analysis

**Fig. 2:**
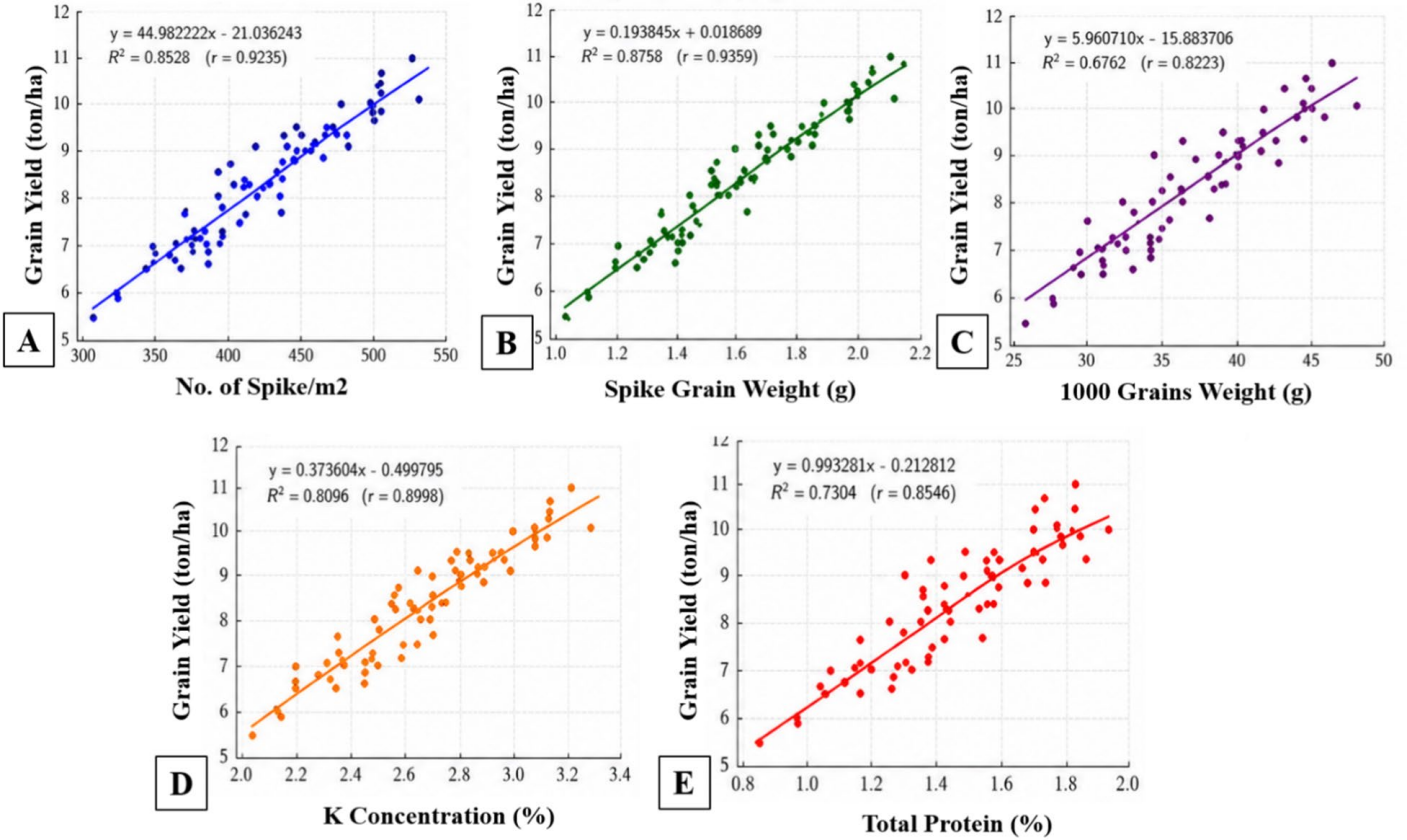
Linear regression relationships between grain yield and its major yield components and grain quality traits in wheat. Regression analyses were performed between grain yield and (A) number of spikes m^−2^, (B) spike grain weight, (C) 1000-grain weight, (D) grain potassium concentration, and (E) grain protein content. The regression equations, coefficients of determination (R^2^), and correlation coefficients (r) are presented in each panel.

To further evaluate the relationships between grain yield and its major yield components and grain quality traits, simple linear regression analyses were performed (Fig. 2). Grain yield exhibited significant positive linear relationships with all evaluated variables. The strongest relationship was observed between grain yield and the number of spikes/m^2^ (R^2^ = 0.8528, *r* = 0.9235), indicating that spike density was the principal determinant of grain yield under the experimental conditions. Grain yield was also highly correlated with spike grain weight (R^2^ = 0.8758, *r* = 0.9359), suggesting that increases in spike productivity contributed substantially to yield improvement.

Significant positive relationships were likewise observed between grain yield and 1000-grain weight (R^2^ = 0.6762, *r* = 0.8223), grain potassium concentration (R^2^ = 0.8096, *r* = 0.8998), and grain protein content (R^2^ = 0.7304, *r* = 0.8546). The relatively high coefficients of determination indicate that improvements in yield components and grain nutritional quality were closely associated with increased grain yield. Overall, the regression analysis confirmed that both yield components and grain nutrient status contributed positively to wheat productivity and supported the treatment effects observed in the factorial analysis.

## Discussion

The results of the two growing seasons demonstrated that wheat performance was strongly influenced by both the potassium source and cobalt application, supporting our hypothesis that potassium source substitution affects crop productivity. The superior performance of the 100% K_2_SO_4_ + Co treatment indicates that adequate potassium supply combined with cobalt nutrition promotes vegetative growth, spike development, and grain production more effectively than potassium supplied solely as KCl. Cobalt likely enhanced these responses through its beneficial effects on nutrient uptake, enzymatic activity, and photosynthetic efficiency, thereby improving overall plant vigor and biomass production, as previously reported by Kandil and El-Maghraby^25^.

In contrast, increasing the proportion of KCl progressively reduced vegetative growth, suggesting that complete replacement of K_2_SO_4_ with KCl is not optimal under the conditions of the present study. This response may be attributed not only to the absence of sulfur in KCl but also to the greater chloride input associated with higher KCl application rates, which may adversely affect nutrient balance and plant metabolism. Conversely, K_2_SO_4_ supplied both potassium and sulfur, the latter being essential for chlorophyll formation, enzyme activation, and the synthesis of sulfur-containing amino acids and proteins. Therefore, the superior performance of the K_2_SO_4_ treatments likely resulted from the combined nutritional benefits of potassium and sulfur rather than potassium alone. These findings are consistent with previous studies reporting greater biomass production and grain yield with potassium sulfate than with potassium chloride, particularly in crops sensitive to chloride or grown under arid conditions^39^.

The present findings are in agreement with previous studies demonstrating that potassium source influences wheat productivity and that blending K_2_SO_4_ with KCl can reduce fertilizer costs without substantially compromising crop performance. Umar et al. ^40^ similarly reported superior wheat growth under K_2_SO_4_ fertilization, while Krauss^41^ suggested that combining K_2_SO_4_ with KCl represents a practical and economical fertilization strategy. The beneficial effects of cobalt observed in the present study are also consistent with earlier reports showing that cobalt enhances plant growth and yield through improvements in physiological processes such as nutrient utilization, chlorophyll development, and enzymatic activity. Comparable responses have been reported in wheat^4,42^, fenugreek^43^, sweet fennel^44^, and rice^42,45^, indicating that the positive effects of cobalt are not crop-specific but represent a broader enhancement of plant performance under appropriate nutritional management. Collectively, these studies support the potential of integrating balanced potassium fertilization with cobalt supplementation to improve crop productivity while optimizing fertilizer use efficiency.

The findings demonstrate that both potassium source and cobalt supplementation had significant effects on wheat yield components and total grain yield, with the combination of 100% K_2_SO_4_ and cobalt (10 mg L^−1^) achieving the highest performance across all measured traits. Independent of potassium source, cobalt application consistently improved yield parameters, confirming its beneficial role in crop productivity. These results are consistent with previous studies highlighting cobalt’s contribution to enhancing growth and yield through its involvement in key physiological and biochemical processes, including nitrogen fixation, enzyme activation, and the delay of leaf senescence^25,46^.

Wheat grains fertilized with potassium sulfate (K_2_SO_4_) showed superior concentrations of both macro- and micronutrients, and these values were further enhanced by cobalt supplementation compared with K_2_SO_4_ alone. Similarly, potassium chloride (KCl) fertilization also benefited from cobalt addition, producing higher nutrient concentrations than KCl without cobalt. However, in both cases, K_2_SO_4_ treatments—whether with or without cobalt—outperformed their corresponding KCl treatments, with the greatest improvements observed under the combined application of K_2_SO_4_ and cobalt. As the proportion of potassium chloride (KCl) increased, there was a notable decline in these nutrient concentrations, with the lowest values observed in the 100% KCl treatment. This pattern agrees with previous studies reporting that sulfate- or silicate-based potassium sources often enhance nutrient profiles and crop performance compared with KCl, which may negatively impact plant nutrition through chloride toxicity or unfavorable nutrient interactions^9,47^. Similar findings were reported by Nadia Gad et al. ^48^, who found that cobalt at 12 mg L^−1^ improved growth, yield, and the nutritional status of broad bean in terms of N, P, K, Mn, Zn, and Cu. Likewise, Gad and Abd El-Hady^49^ and Wa Lwalaba et al. ^50^ observed that cobalt addition at 12.5 mg L^−1^ significantly improved most macro- and micronutrient concentrations across potassium treatments, with the exception of Fe. The observed decline in Fe under cobalt supplementation supports earlier reports of antagonistic interactions between Fe and Co, where increased cobalt levels reduce iron content, photosynthesis, and chlorophyll in crops such as tomato and soybean.

Application of cobalt at 10 mg L^−1^ improved the nutritional profile of wheat grains across nearly all potassium treatments, indicating a synergistic interaction between cobalt and potassium—particularly potassium sulfate in enhancing nutrient uptake and accumulation. However, an important exception was observed under the 100% K_2_SO_4_ + cobalt treatment, where grain cobalt concentration rose sharply to 3.9 mg L^−1^, substantially higher than in all other treatments. However, the grain cobalt concentration recorded under the 100% K_2_SO_4_ + Co treatment exceeded the commonly recommended limit of approximately 1 mg kg^−1^ for wheat grain. Although cobalt application improved growth, yield, and grain nutritional quality, this finding indicates that caution is required when applying cobalt fertilizers because excessive accumulation in edible grains may pose risks to human and animal health^51,52^. Therefore, optimization of cobalt application rates should be considered in future studies to maximize agronomic benefits while ensuring food safety.

The findings highlight the significant influence of both potassium source and cobalt supplementation on the biochemical composition of wheat grains. This agrees with earlier reports showing that cobalt, through its role in enzymatic regulation and nitrogen metabolism, promotes protein synthesis and carbohydrate accumulation^18,53^. As a trace element, cobalt enhances biological nitrogen fixation and overall metabolic activity, which likely contributes to the observed increases in protein content and soluble sugars^54,55^. The choice of potassium source also proved critical for grain quality, with K_2_SO_4_-based treatments consistently outperforming KCl. The superior performance of the K_2_SO_4_ treatments may be attributed not only to the potassium supplied but also to the accompanying sulfur. At the applied rate of 238 kg ha^−1^, K_2_SO_4_ (18% S) supplied approximately 42.8 kg S ha^−1^, which likely contributed to improved protein synthesis, the formation of sulfur-containing amino acids, enzyme activity, and overall nitrogen-use efficiency. Consequently, the greater grain yield and quality observed with K_2_SO_4_ compared with KCl probably resulted from the combined nutritional effects of potassium and sulfur rather than potassium alone. This interpretation is supported by previous studies reporting enhanced protein accumulation and carbohydrate metabolism following sulfur fertilization^56,57^. In contrast, chloride ions supplied by KCl may inhibit nitrate uptake and metabolism^9^, leading to reduced protein biosynthesis and lower sugar accumulation^9^. These observations are consistent with previous work: Gad et al. ^24^ found that 10 ppm cobalt improved growth, yield, mineral composition, and biochemical constituents of faba bean; Bonfim-Silva et al. ^58^ reported that potassium stimulates nitrogen absorption and amino acid translocation to wheat grains, favoring gluten and protamine synthesis; Bhattarai and Swarnima^59^ noted that potassium enhances protein, starch, vitamin C, and soluble solids in grains, tubers, and fruits; and Xi et al. ^60^ demonstrated that K_2_SO_4_ promotes amino acid formation in tea.

The regression analysis further supports the conclusion that improvements in grain yield were closely associated with enhanced yield components and grain nutritional status. The strong positive relationships between grain yield and the number of spikes/m^2^, spike grain weight, and 1000-grain weight indicate that increased productivity resulted primarily from improved sink capacity and grain filling^61^. Likewise, the positive associations between grain yield, grain potassium concentration, and protein content suggest that improved potassium nutrition enhanced nutrient utilization and nitrogen metabolism, contributing simultaneously to higher yield and improved grain quality. These relationships reinforce the conclusion that the combined application of potassium and cobalt improved wheat productivity through coordinated effects on both yield formation and nutrient accumulation.

Overall, the significant interaction between potassium source and cobalt application demonstrated that cobalt enhanced wheat growth, yield, grain quality, and nutrient accumulation across all potassium treatments, with the greatest improvements observed in treatments containing higher proportions of K_2_SO_4_. Although the 100% K_2_SO_4_ treatment consistently produced the best agronomic performance, the present study demonstrated that partial substitution of K_2_SO_4_ with the less expensive KCl maintained satisfactory crop growth, yield, and grain quality, whereas complete replacement with KCl resulted in significant reductions in most measured parameters. Furthermore, cobalt application partially alleviated the adverse effects associated with increasing KCl proportions, improving crop performance and grain nutritional quality. These findings indicate that integrating cobalt application with partial substitution of K_2_SO_4_ by KCl offers a practical, cost-effective, and sustainable fertilization strategy for wheat production under arid soil conditions, while complete replacement of K_2_SO_4_ with KCl cannot be recommended based on the results of the present study.

## Conclusions

The findings of this study clearly demonstrate that the source of potassium fertilizer and the application of cobalt significantly influence wheat growth, yield, and grain quality under arid soil conditions. The application of potassium sulfate (K_2_SO_4_) proved to be more effective than potassium chloride (KCl), particularly when combined with cobalt supplementation at 10 mg L^−1^. The combined use of 100% K_2_SO_4_ and cobalt consistently enhanced vegetative growth, spike characteristics, grain yield, nutrient uptake (especially nitrogen, potassium, and zinc), and key grain constituents such as protein, carbohydrates, starch, and soluble sugars. Conversely, increasing proportions of KCl led to notable declines in all measured parameters, including nutrient content and grain quality, highlighting the detrimental impact of chloride-based potassium fertilization. While cobalt application independently improved several traits, its synergistic effect with K_2_SO_4_ was markedly superior, suggesting an interaction that enhances physiological performance and metabolic processes in wheat plants. Overall, these results advocate for the integrated use of K_2_SO_4_ and cobalt as an agronomically and environmentally sound strategy for optimizing wheat productivity and grain nutritional quality, especially in saline or nutrient-deficient soils. Future research should evaluate a wider range of cobalt application rates under different soil and environmental conditions to determine the optimum dose that enhances wheat productivity while maintaining grain cobalt concentrations within safe limits for food and feed.

## Data Availability

Data is provided within the manuscript.
